# Identification of two methylated fragments of an SDC2 CpG island using a sliding window technique for early detection of colorectal cancer

**DOI:** 10.1002/2211-5463.13180

**Published:** 2021-06-07

**Authors:** Ruibin Li, Bing Qu, Kangkang Wan, Changming Lu, Tingting Li, Fuxiang Zhou, Jun Lin

**Affiliations:** ^1^ Hubei Key Laboratory of Tumor Biological Behavior Hubei Cancer Clinical Study Center Zhongnan Hospital of Wuhan University China; ^2^ Department of Radiation and Medical Oncology Zhongnan Hospital of Wuhan University Wuhan China; ^3^ Department of Reproductive Medicine Center Renmin Hospital of Wuhan University & Hubei Clinic Research Center for Assisted Reproductive Technology and Embryonic Development China; ^4^ Department of Science and Education China Resources & WISCO General Hospital, Wuhan University of Science and Technology Wuhan China; ^5^ Wuhan Ammunition Life‐tech Company, Ltd. China; ^6^ Department of Gastroenterology Zhongnan Hospital of Wuhan University China

**Keywords:** biomarkers, colorectal cancer, early detection, methylation, SDC2

## Abstract

Colorectal cancer (CRC) is one of the most common cancer types globally with a 5‐year survival rate of < 50% in China. Aberrant DNA methylation is one of the hallmarks of tumor initiation, progression, and metastasis. Here, we investigated the clinical performance of two differentially methylated regions (DMRs) in SDC2 CpG islands for the detection of CRC. A sliding window technique was used to identify the DMRs, and methylation‐specific PCR assay was used to assess the DMRs in 198 CRC samples and 54 normal controls. Two DMRs (DMR2 and DMR5) were identified using The Cancer Genome Atlas (TCGA) data, and the hypermethylation of DMR2 and DMR5 was detected in 90.91% (180/198) and 89.90% (178/198) of CRC samples, respectively. When combining DMR2 and DMR5, the sensitivity for CRC detection was 94.4% higher than that of DMR2 or DMR5 alone. Based on the above results, we propose using DMR2 and DMR5 as a sensitive biomarker to detect CRC.

## Graphical abstract

We identified two differentially methylated regions (DMRs) between colorectal cancer samples and normal controls using a sliding window method. Receiver operating characteristic (ROC) analysis suggests these two DMRs have potential for distinguishing cancers from normal controls using both the TCGA dataset and our validation set.

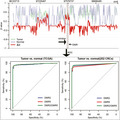

**Abbreviations**
AUC: Area under the ROC curve
CIMP: CpG island methylation phenotype
CRC: Colorectal cancer
DMR: Differentially methylated region
DNMT: DNA methyltransferase
FFPE: Formalin‐fixed paraffin‐embedded
MSP: Methylation‐specific PCR
MSPR: MSP region
QPCR: Quantitative Real‐time PCR
ROC: Receiver operating characteristic
SDC2: Syndecan 2
TCGA: The Cancer Genome Atlas
TET: Ten‐eleven translocation cytosine dioxygenase

Colorectal cancer (CRC) is one of the most common cancer types in the world [1] with a 5‐year survival rate of < 50% in China [2]. More than 1.9 million CRC cases were newly diagnosed, and almost one million deaths were attributable to CRC in 2020. Overall, CRC incidence and mortality ranked third and second of all cancers, respectively [1]. One of the reasons for the high mortality of CRC is that only a minority of CRC patients are detected at an early stage [3]. Although existing colonoscopy screening could effectively uncover CRC, the inconvenience and invasiveness of colonoscopy make it difficult to be performed often [4]. Several diagnostic tools have been developed to facilitate the early detection of CRC, including fecal occult blood detection (FOBT) and fecal immunochemistry test (FIT). However, these methods are limited by their low sensitivity and specificity in detecting CRC and advanced adenoma [5]. Thus, noninvasive methods with high sensitivity and specificity are urgently needed for the early detection of colorectal neoplasia.

Aberrant DNA methylation, such as hypermethylated promoters of tumor suppressors and hypomethylated intergenic regions, is one of the hallmarks of tumor initiation, progression, and metastasis [6] which can occur very early in cancer pathogenesis. Abnormalities in DNA methylation usually refer to the modification of the C5 position of the cytosine ring, typically in a CpG site, by adding or removing methyl groups. DNA methyltransferases (DNMTs) are the main enzymes that transfer CH3 groups from S‐adenosyl‐l‐methionine to cytosine to form 5‐methylcytosine [7]. DNA demethylation involves multiple mechanisms, including passive and active processes. The passive process is associated with aberrant DNA replication, while the active process is typically regulated by ten‐eleven translocation cytosine dioxygenases (TETs) and thymine DNA glycosylase [8, 9, 10]. DNA methylation is known to occur abnormally during the early stages of many cancers [11]. These aberrantly methylated regions are promising targets for the development of powerful diagnostic biomarkers. Several methylated markers have been developed for the early detection of CRC, including Epi pro colon (with methylated SEPT9 as a detection target [12]) and the Exact Sciences’ multi‐target stool DNA test (with methylated BMP3 and NDRG4 as two of its targets [13]). However, the sensitivity and specificity of different markers vary widely, and none of them have a sensitivity exceeding 90% [14]. Syndecan 2 (SDC2) was previously reported to be associated with the cell signal transduction, migration, and proliferation of CRC [15, 16, 17, 18]. Hypermethylation of SDC2 was detected at a high frequency in the blood and stool of patients with CRC [16, 17]. However, the sensitivity of CRC detection in stool samples is only 80%–90% [19, 20].

The genomic location of methylation‐based biomarkers is an important feature that plays a significant but overlooked role [21]. The promoter of a detection target may contain more than one CpG island, and not all islands are functionally equivalent [22, 23], or only one CpG island may cover a long base region. However, the methylation status of neighboring CpG sites often tends to be highly similar [24]. Several tools have been developed to identify differentially methylated regions (DMRs) [25, 26, 27], but none have been designed to specifically identify cancer diagnostic biomarkers. These methods mainly focused on detecting larger methylation differences between phenotypes, and the size of identified DMRs usually exceeds the limited length of the PCR amplicon [22, 28]. In addition, these detected DMRs often do not show optimal sensitivity or specificity in distinguishing cancers from normal controls, because of their different algorithm objects [29], and different targeted regions usually could represent different detection performances.

In this study, we developed an alternative approach to improve the sensitivity of SDC2 for CRC detection. We first separated the genomic sequences into multiple bins by setting different window sizes and then determined the differential methylation values between tumor and normal samples defined as delta β, based on the methylation levels of the CpGs in each bin. The delta β values were used to distinguish CRCs from normal controls to identify the optimal bins that could serve as candidate DMR biomarkers. First, the genomic location of the CpG island in SDC2 was determined using the MEXPRESS website. Five potential DMRs in the CpG island were identified using our custom window sliding approach that was applied to methylation data from The Cancer Genome Atlas (TCGA) CRC dataset, and two nonoverlapping DMRs were selected as the amplification targets for methylation‐specific PCR (MSP) in 198 CRC tissue samples and 54 normal colon samples. Finally, we evaluated the performance of dual‐DMR targets in the SDC2 CpG islands for the detection of CRC. In addition, we further explored the impact of tumor location on the performance of these two DMRs.

## Materials and methods

### Data preparation

Publicly available CRC methylation data generated using the Illumina Infinium HumanMethylation450k BeadChip kit were downloaded from the TCGA portal (https://portal.gdc.cancer.gov/). The β values of DNA methylation probes were then used for the comparison of 45 tumors and matching adjacent normal samples in the TCGA CRC dataset. Samples resected from the cecum, ascending colon, and hepatic flexure of the colon were classified as the right‐sided tumor, while samples from the descending colon, sigmoid colon, and rectosigmoid junction were grouped as left‐sided tumors. Samples not classified to the right‐ or left‐sided groups were defined as the other groups (Table S1).

### Identification of DMRs

CpG islands of SDC2 were identified from Mexpress [30] by entering the gene symbol ‘SDC2’ and selecting the cancer type as colon adenocarcinoma. The UCSC Xena browser was used to analyze the genomic context of CpG islands. We used the sliding window approach [31] to determine the DMRs. Briefly, the probes within 2 kb upstream of TSS, gene body, and 0.2 kb downstream of SDC2 were selected and sorted by their coordinates. With a predefined window size and step size, these probes were separated into multiple fragments with overlapping regions of equal length. Here we set window sizes ranging from 2 to 10 CpG sites, with a fixed step size of 1 CpG. The mean methylation level of the probes within each sliding window was calculated as the methylation level of the region. Finally, Δβ values were defined as the different methylation levels of CpG regions between tumor and normal samples.

### Patients and samples

Patients and samples with information on age, sex, and clinical diagnosis were selected. Samples with confirmed colonoscopy and/or pathologic diagnosis were considered to have CRC. Normal samples refer to tumor‐adjacent tissues. Patients with incomplete information, including an incomplete history of CRC surgery, history of chemotherapy, or any other treatment, were excluded. For the collection of stool samples, we also excluded patients who had familial or hereditary colorectal adenomas/tumors, underwent any form of colorectal invasive procedure, or took laxative bowel preparation for < 1 week before endoscopic or tumor resection. Non‐CRC patients who received any chemotherapy within the last 6 months were also excluded. This project was approved by the ethics committee of Zhongnan Hospital of Wuhan University before the start of the clinical trial (No.2019099). All participants signed the informed consent before tissue collection and were informed about the usage of their samples and the test results. The use of human samples complied with the standards stipulated in the Declaration of Helsinki.

### DNA isolation of tissue samples

Tumor tissue samples were collected from formalin‐fixed paraffin‐embedded (FFPE) specimens, and genomic DNA was extracted using the UnigeneDx FFEE DNA extraction kit according to the manufacturer’s instructions. Target genes in tumor tissues were captured using previously reported technology with some modifications [32]. We redesigned the capture probe for the target gene as we focused on different regions compared with the reference. The amplification regions in this study covered two CpG sites in the 5′ regulatory region and three other CpG sites in the gene body (+401 → +1983 bp), which captured a larger genomic region and contained more CpG sites.

### Bisulfite conversion

Tissue‐derived genomic DNA was chemically modified by sodium bisulfite to convert unmethylated cytosine to uracil while leaving methylated cytosine unchanged. Briefly, DNA was incubated at 98 °C for 10 min, then treated with sodium bisulfite at 64 °C for 1 h. Bisulfite‐treated DNA was added to Biocomma spin columns (Biocomma, China) for purification and then centrifuged at 13 400 *g* for 30 s. Next, DNA was washed once and desulfonated at room temperature for 15 min. DNA was subsequently washed twice and eluted with 40 µL of TE buffer. The eluted DNA was either used immediately for Quantitative Real‐time PCR (QPCR) analysis or stored at −20 °C for further use.

### Methylation‐specific PCR

Methylation‐specific PCR was used to determine the methylation status of SDC2 in normal and tumor tissue DNA, and β‐actin was used as an internal control [33, 34]. Specific primers and probes for the target region of DMR2 and DMR5 were designed as shown in Table 1. PCR was performed using a High Affinity Hotstart Taq Polymerase kit (TIANGEN). Bisulfite‐converted tissue or stool DNA (5 µL) was added as a template. PCRs was performed on an ABI 7500 instrument under the following cycling conditions: 95 °C for 5 min, 95 °C for 15 s, and 45 cycles at 60 °C for 30 s. We used the cycle threshold (Ct) value to determine the methylation status of SDC2, and the values for tissue samples were considered ‘invalid’ if the ACTB Ct was > 36.00 and methylated SDC2 was considered ‘detected’ if the Ct values were < 45.00. For samples with no amplification curve of the MSP that occurred after 45 cycles, the Ct value was assigned as 45.00. Three MSP replicates were performed for each sample, and the average Ct value was used for further analysis.

**Table 1:** Primers and probes used in this study.

Name	Primer/Probe sequences (5′‐3′)	Description
MSPR1_F	CGAGTTTGAGTCGTAATCGTTGC	MSP region 1 forward primer
MSPR1_R	GCAAACCACCAAACCCAAAATAAAC	MSP region 1 reverse primer
MSPR1_P	CTACTCCCAACCGCTACTTACAACC	MSP region 1 probe
MSPR2_F	CGTCGGTTATTGGATTTTTAG	MSP region 2 forward primer
MSPR2_R	TCTATCCCCCAACGACCAAAC	MSP region 2 reverse primer
MSPR2_P	GCCTCGCCCTACTTACGACACTC	MSP region 2 probe
ACTB_F	CGCAATAAATCTAAACAAACTCC	ACTB forward primer
ACTB_R	AGGTTAGATGGGGGATATGT	ACTB reverse primer
ACTB_P	TCCCAAAACCCCAACACACT	ACTB probe

### Statistical test

The sample size was estimated based on the method described in a previous study [35]. The *P* value was selected as 0.85, based on the existing literature on SDC2 sensitivity for tissue samples. Thus, a total of 195 CRC cases were required. In this study, we collected the complete information of 198 patients who fully met the criteria (Table 2). Bioinformatics and statistical analyses were performed using r version 3.6.1 [36]. Group comparisons of matched normal‐tumor samples were performed for continuous variables using the independent paired *t*‐test, and the Kruskal–Wallis rank test was used for multiple group comparisons. The sensitivity and specificity of the diagnostic risk models were evaluated using the receiver operating characteristic (ROC) curve [37] and quantified by the area under the ROC curve (AUC). All statistical tests were two‐sided, and statistical significance was set at *P* < 0.05.

**Table 2:** Colorectal sample characteristics.

	Tumor	Normal
Number	198	54
Sex		
Male	119	32
Female	79	22
Location		
Right colon	42	15
Left colon	73	25
Rectum	70	14
Others	13	0
Age, median(25%–75%)	63 (55–69)	59 (48–71)
Grade		
G1	20	54
G2	142	
G3	15	
Clinical stage		
Stage I	10	54
Stage II	73	
Stage III	94	
Stage IV	11	
NA	10	

## Results

### Identification of DMRs in SDC2 CpG islands

**Fig. 1:**
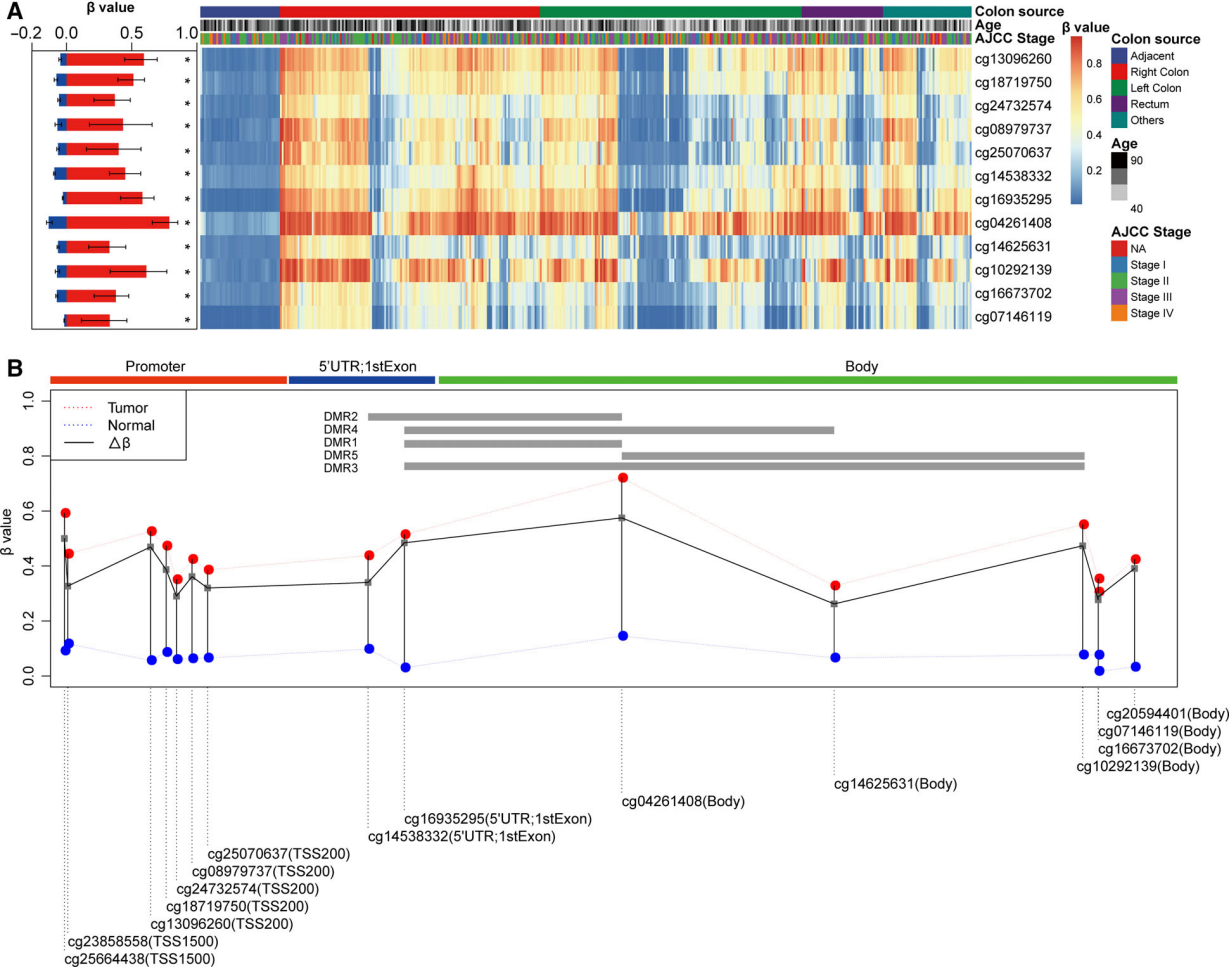
Methylation profile of the SDC2 CpG island in the normal and tumor samples of the TCGA CRC dataset. (A) Methylation levels of 12 CpG sites. (B) Genome structure of the 5 DMRs. Red bars and blue bars in the left panel represent the average methylation level of tumor samples (*n* = 391) and normal samples (*n* = 45), respectively. ‘*’ indicates a significant difference in methylation level between tumor and normal samples. The upper and lower error bars indicate ± SD of the average methylation levels. Wilcox rank sum test was used to estimate the significant difference.

Only one CpG island within SDC2 was identified according to the MEXPRESS website (Table S2). A total of 731 nucleotides of the CpG island from 96493601 bp to 96494331 bp constituted the 5′UTR of the SDC2 gene, 81 nucleotides located upstream 96493601 bp, and 1048 nucleotides downstream 96494331 bp were part of a promoter and one intron of the SDC2 gene, respectively (Table S3). The Illumina Human Methylation 450 K probes covered by CpG islands were extracted from the TCGA portal. Twelve probes located in this region were selected, all of which were hypermethylated in tumor samples compared to normal samples (Fig. 1A). Similarities in β values across multiple CpG sites within the target region indicated that multiple CpG sites in a hypermethylated region could enhance the detection accuracy instead of a single CpG site.

The window sliding approach identified five DMRs, with the largest Δβ values on the SDC2 CpG island (Table 3). The methylation level of CpG site cg04261408 was the highest, and all five DMRs overlapped within this CpG site (Fig. 1B). Based on the coordinates of the five DMRs, we selected DMR2 and DMR5, which did not overlap with each other, as the potential target regions for the next MSP procedure that would help to improve CRC detection performance (Fig. 1B).

**Table 3:** Summary of 5 DMRs in the SDC2 CpG island.

DMRs	Position	Probe ID	Normal (mean ± SD, *n* = 45)	Tumor (mean ± SD, *n* = 391)	Δβ	length
DMR1	Chr8:96494023‐96494448	cg16935295	0.081 ± 0.035	0.676 ± 0.202	0.595	425bp
		cg04261408				
DMR2	Chr8:96493952‐96494448	cg14538332	0.086 ± 0.031	0.599 ± 0.186	0.513	496bp
		cg16935295				
		cg04261408				
DMR3	Chr8:96494023‐96495334	cg16935295	0.074 ± 0.023	0.563 ± 0.188	0.489	1311bp
		cg04261408				
		cg14625631				
		cg10292139				
DMR4	Chr8:96494023‐96494852	cg16935295	0.076 ± 0.024	0.555 ± 0.180	0.479	829bp
		cg04261408				
		cg14625631				
DMR5	Chr8:96494447‐96495334	cg04261408	0.092 ± 0.027	0.569 ± 0.185	0.477	887bp
		cg14625631				
		cg10292139				

### Methylation profile of DMR2 and DMR5

**Fig. 2:**
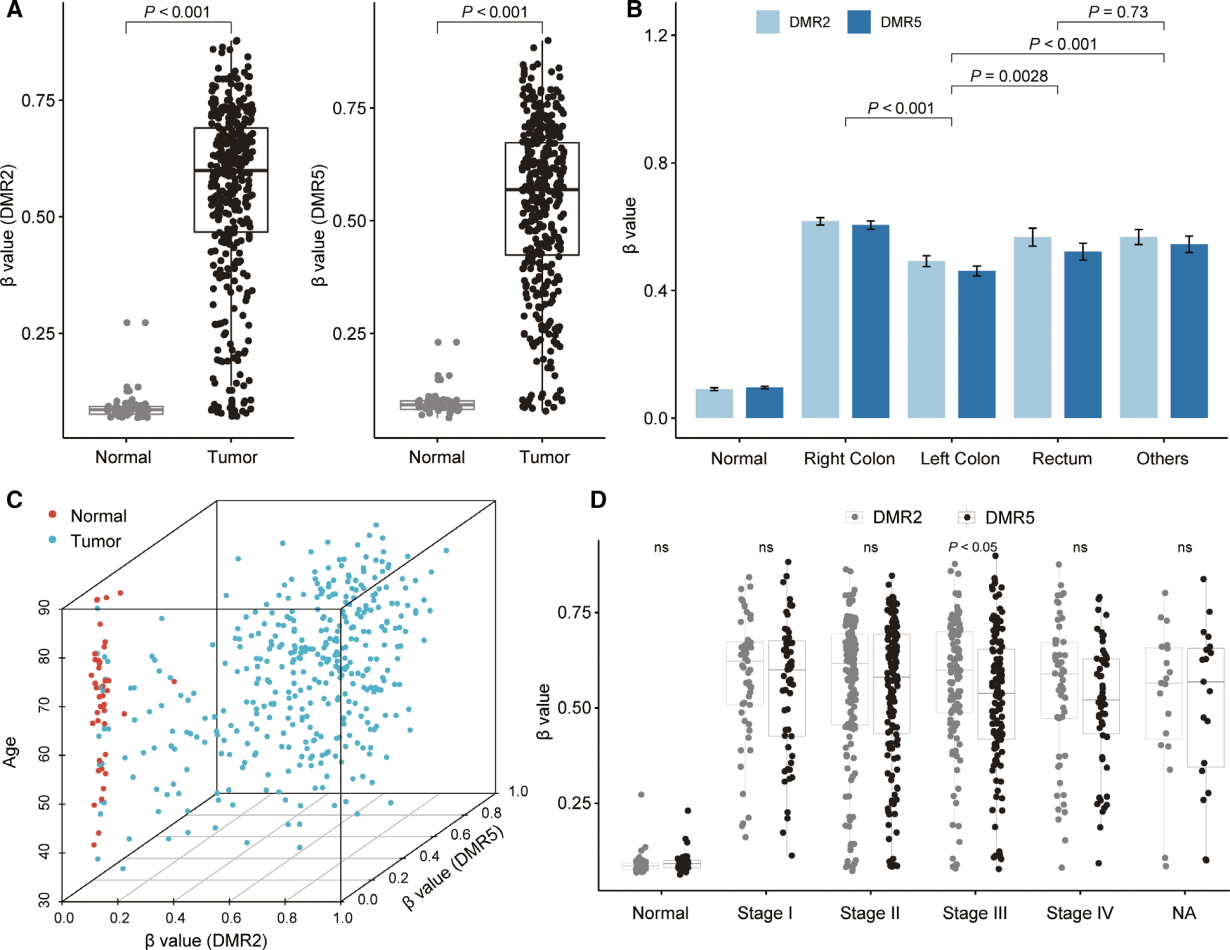
Methylation levels of DMR2 and DMR5 in CRC tissues and normal colon tissues. (A) Methylation levels of DMR2 and DMR5 in 45 normal and 391 tumor samples. (B) Methylation levels of CRC samples from different colon sites. (C) 3‐D scatter plot showing the correlation of β values of DMR2 and DMR5 with patient age. (D) β values of DMR2 and DMR5 in the different stages of CRCs. The error bars in b represent ± SD range. ‘ns’ means not significant. Wilcox rank sum test was used to estimate the significant difference.

Further analysis of the methylation profile of DMR2 and DMR5 from the TCGA CRC dataset showed a significantly higher level of methylation in CRC tissues than in normal tissues, and the beta value was < 0.3 across all normal samples (Fig. 2A). The frequency of DMR2 and DMR5 methylation was also significantly different in CRC of different colon locations. The left‐sided tumors showed lower frequency methylation than right‐sided tumors and the rectum (Fig. 2B, Table S4), indicating a highly heterogeneous methylation status of SDC2 CpG islands across different tumor locations.

Age is a high‐risk factor for genome DNA methylation, and many tumor suppressor genes have been reported to be age‐dependent hypermethylated genes. However, the methylation of DMR2 and DMR5 did not show a strong correlation with age (*R* = 0.064 and 0.11, Fig. 2C). In samples from different pathological stages, the methylation levels of DMR2 and DMR5 were significantly higher than in normal samples (Fig. 2D). This result agrees with that of previous studies, showing that SDC2 was highly methylated in the early stage of CRC [17, 19]. In addition, we found that DMR5 showed a lower methylation level than that of DMR2 in Stage III samples (mean β: 0.52, 0.56, Fig. 2D), which might imply a bias in the performance of advanced CRC detection.

### Identification of DMR2 and DMR5 in CRC tissues

**Fig. 3:**
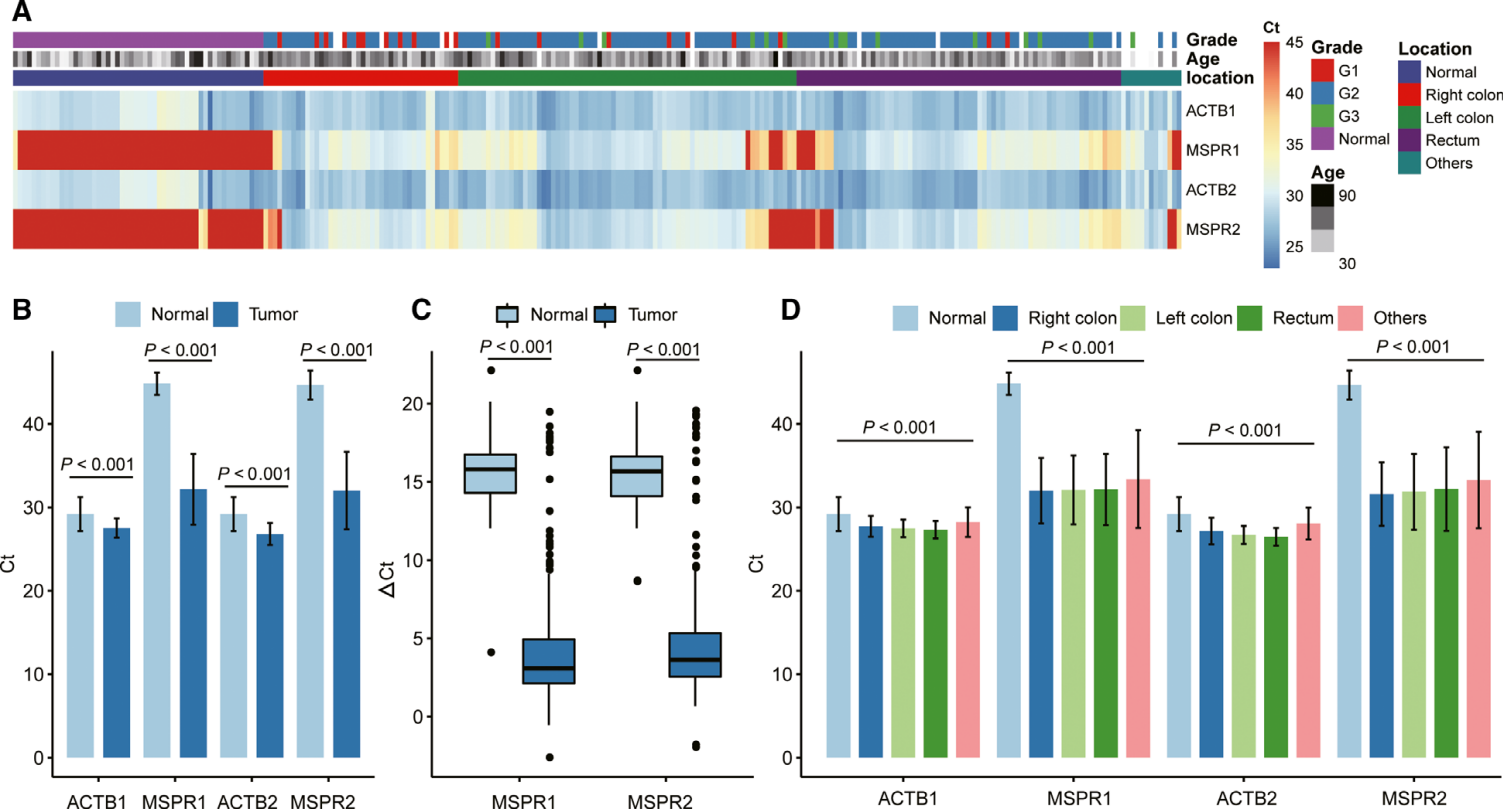
Methylation profiles of MSPR1 and MSPR2 in 198 colorectal tissue samples and 54 normal samples. (A) Heat map presentation associated with the methylation profile in normal (*n* = 54) and tumor samples (*n* = 198). (B) The average methylation levels of MSPR1 and MSPR2 in normal (*n* = 54) and tumor samples (*n* = 198). (C) Boxplots showing ∆Ct values of MSPR1 and MSPR2. (D) The average methylation levels of MSPR1 and MSPR2 in different colon sites of CRC samples. The error bars in B and D represent ± SD range. For pane B and C, the *P* values was estimated by Wilcoxon rank sum test. For panel D, Kruskal–Wallis rank sum test was performed.

Two regions of DMR2 and DMR5 (herein termed MSPR1 and MSPR2) were selected as targets for MSP to quantify the methylation levels of the SDC2 gene in 198 colorectal tissues and 54 normal tissues. The methylation profiles across 252 samples are shown in Fig. 3A, and we can see that most of the tumor samples showed higher methylation levels at MSPR1 and MSPR2 than normal samples (90.91% vs. 1.85% and 89.90% vs. 3.70%). However, a few fractions of left‐sided and rectal tumors (8.4%) presented with lower methylation status, which was consistent with the results obtained from the TCGA data (see Fig. 2B). Median methylation levels of MSPR1 and MSPR2 in normal samples were both 45.00 (interquartile 45.00–45.00), while for tumor samples they were 30.97 (interquartile 29.60–33.02) and 30.71 (interquartile 29.20–32.86; Fig. 3B). ∆Ct, which was defined as the difference between the Ct values of the targets (MSPR1 and MSPR2) and the reference gene ACTB also varied significantly among normal tissues and tumor samples (Fig. 3C). All the different CRC locations showed high‐frequency methylation compared to normal samples, with no significant difference in values across CRC locations (Fig. 3D, Table S5).

### Performance of the 2‐DMR based diagnostic prediction model for CRC

**Fig. 4:**
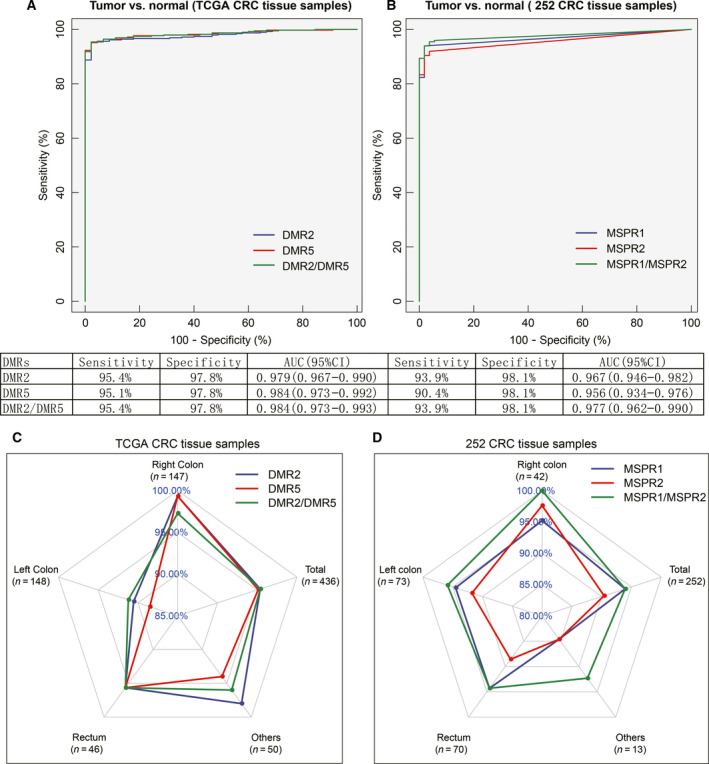
Diagnostic performance of 2‐DMRs methylation levels in TCGA CRC cohort and 252 CRC tissue cohort. (A) ROC curves for DMR2 and DMR5 in detecting TCGA CRC (*n* = 436). (B) ROC curves for MSPR1 and MSPR2 in detecting 252 CRC. (C) The sensitivity of DMR2 and DMR5 at detecting different colon sites of CRC from TCGA dataset. (D) The sensitivity of MSPR1 and MSPR2 at detecting different colon site of CRC from our dataset.

**Fig. 5:**
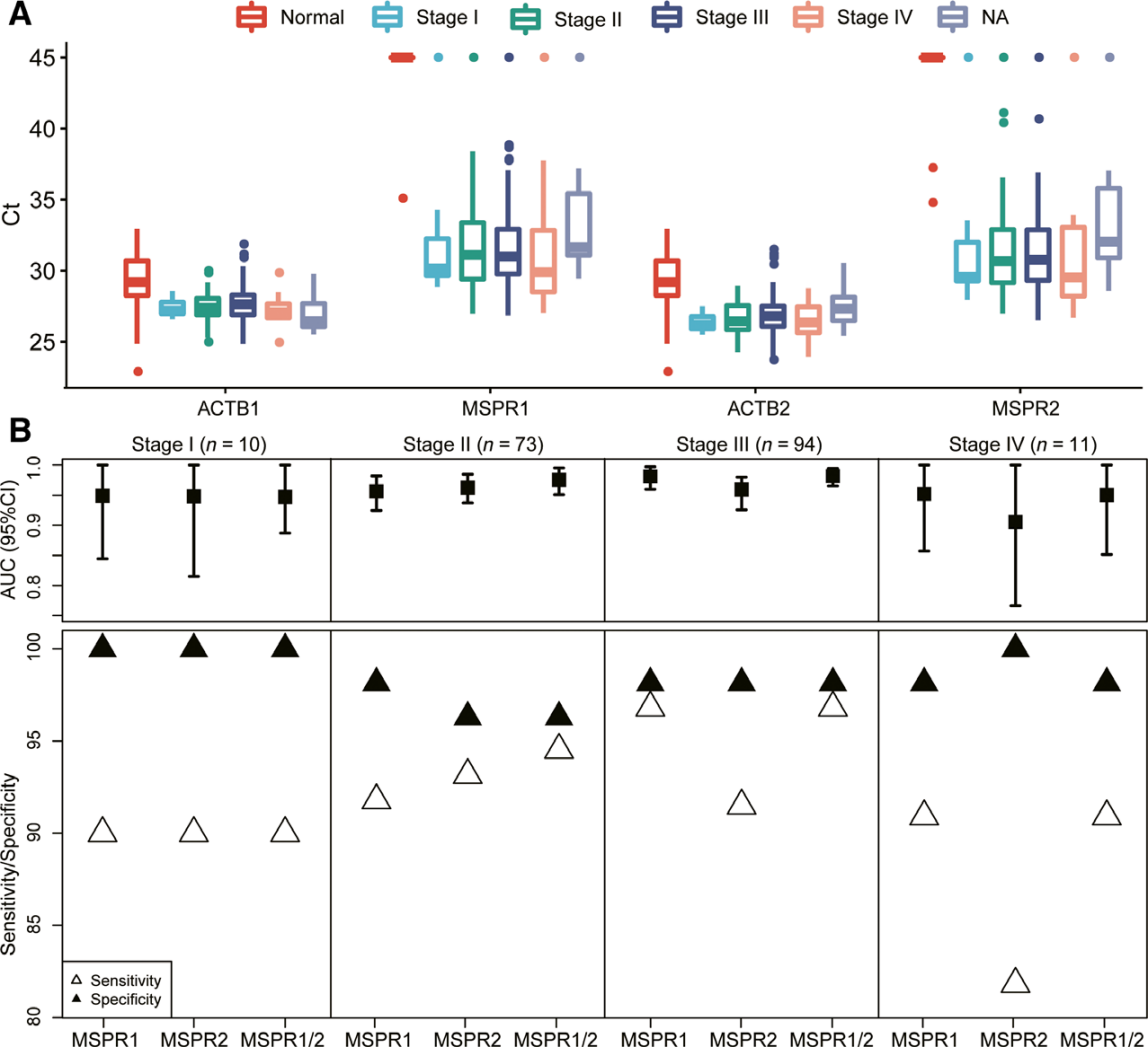
Performance of MSPR1 and MSPR2 in detecting CRCs according to clinical stages. (A) Ct values of MSPR1 and MSPR2 at different stages of CRCs (*n* = 198) and normal controls (*n* = 54). (B) Sensitivity, specificity, and AUC of MSPR1 and MSPR2 for detecting CRC at different stages. NA: not available. Error bars in panel b indicate 95% confidance interval of AUC values.

A two‐class prediction model was developed using the logistic binomial regression method, based on 2‐DMR methylation levels [38]. ROC curves were then constructed to evaluate the performance of methylated DMR2 and DMR5 for detecting colorectal samples. The AUC for DMR2 alone and DMR5 alone was 0.979 (95% CI: 0.967–0.990) and 0.984 (95% CI: 0.973–0.992) in the TCGA CRC cohort. No difference in AUC was found when combining DMR2 and DMR5 (Fig. 4A). However, for our 252 CRC samples, the methylated MSPR1 and MSPR2 panels improved the AUC from 0.967 (95% CI: 0.946–0.982) and 0.956 (95% CI: 0.934–0.976) to 0.977 (95% CI: 0.962–0.990; Fig. 4B). For CRCs from different tumor locations, we further evaluated the performance of DMR2 and DMR5 in discriminating tumors from normal samples. Interestingly, we found a higher sensitivity for DMR2 than DMR5 in left‐sided CRCs and other types, despite the lack of a difference in the sensitivity of all samples for both DMRs (Fig. 4C). Moreover, it was confirmed in our 198 CRC and 54 control cohorts that the sensitivities of MSPR1 were higher in left‐sided and rectal CRCs than of MSPR2 (Fig. 4D). This result suggested a complementary methylation pattern of the two DMRs, which potentially improved the performance of the diagnostic prediction model for CRC. We also found higher methylation levels of DMR2 and DMR5 at all stages of CRC than in normal controls (Fig. 5A), which is in line with other studies [17, 19]. The sensitivity and specificity of both DMRs for various stages reached above 90% (Fig. 5B), reflecting their good performance in the early stages (I–II) and advanced stages (III–IV) of CRCs. Comparisons between DMR2/DMR5 and the currently available methods are listed in Table 4.

**Table 4:** Performance of different CRC screening tests.

Methods	Sensitivity	Specificity
Colonoscopy	>95%	~100%
gFOBT	33%–75%	80%–95%
FIT	50%–95%	80%–95%
DMR2/DMR5	93.90%	98.10%

## Discussion

DNA methylation‐based biomarkers are promising candidates for the detection of CRC, but so far, only a few have been commercialized. One possible reason for this is the obscure relationship between the methylation of DMRs and their precise genomic locations. In this study, we used a custom window sliding method to identify the optimal DMRs within the SDC2 CpG island between tumor and normal samples. Since the methylation status of adjacent CpG sites in one CpG island was tightly relevant [39], it was reasonable to treat the region encompassed by probes as a whole detection target. All of the five optimal DMRs identified by our method showed significantly hypermethylated status in tumor samples compared to normal samples, with cg04261408 showing the highest methylation level among the 12 CpG sites. For CRCs derived from different colon locations, the left‐sided samples showed the lowest frequent methylation status in both DMR2 and DRM5, which were then selected as the amplified regions of the MSP. The high heterogeneity of CRC is reflected in its complex molecular phenotypes, and the CpG island methylation phenotype (CIMP) is one of the three major methods used to identify the disease. The CIMP subgroup is characterized by vast hypermethylated CpG sites, which can cause the silencing of many genes, including tumor suppressors or other related genes [40]. Previous studies have reported that right‐sided tumors were more frequently CIMP‐high than left‐sided tumors [41, 42], which is consistent with our results that DMR2 and DMR5 presented higher levels of methylation in right‐sided CRC. Based on this, we hypothesized that methylated SDC2 may contribute to the definition of high CIMP. Previous studies have demonstrated that SDC2 methylation is independent of patients’ clinical features, including sex and age, and that hypermethylation can occur in early stage CRCs [43]. In this study, we also found no strong correlation between DMR2/DMR5 methylation and patient age. Although one CpG site (cg25070637) in the promoter of SDC2 was reported to be significantly associated with age [44], this site did not overlap with DMR2/DMR5 according to the genomic locations. It has been widely accepted that DNA methylation is associated with age via silencing gene expression; however, the elevated promoter methylation of SDC2 is not obviously correlated with its expression [45]. This might, on the other hand, indicate the weak correlation between DMR2/DMR5 methylation and patient age.

The sensitivity for detecting CRC by DMR2 alone and DMR5 alone was 95.4% and 95.1% in the TCGA CRC cohort, respectively. The combination of DMR2 and DMR5 improved the overall sensitivity to 95.4%. No difference in specificity was found irrespective of the combination of DMR2 and DMR5 or alone (Fig. 4A). The sensitivity of MSPR1, MSPR2 alone, and the combination of the two were 93.9%, 90.4%, and 93.9%, respectively. The results of this study were higher than those of Han et al. [19] and Niu et al. [20], but the specificities were comparable. Our study suggested that a panel of multiple DMRs of SDC2 as detection targets, performed much better than a single biomarker.

The current study utilized a sliding window method to detect potential DMRs, and its good performance in CRC diagnosis was validated by the MSP array. However, there are some limitations to this study. First, we averaged all CpG sites in a given region, which might not be reasonable for some DMRs, due to the nonuniform distribution of CpG sites [46]. In addition, the Infinium Methylation 450 K array only covers approximately 1.5% of the total genomic CpG sites and is mainly concentrated on gene promoters and bodies, which could hinder us from obtaining better DMRs [47].

Notably, a relatively weaker performance of DMR5 in left‐sided tumor detection than DMR2 was found, which was confirmed by our MSP data of 252 samples. However, this result was not observed in other studies [19, 20], which might be due to methodological problems such as amplicon primers and DNA extraction methods that masked the methylation level differences between the left and right colon. Nonetheless, the more frequent methylation status of all 12 probes in the CpG island was found in right‐sided tumors, and no significant difference was found between the right‐ and left‐sided normal samples, suggesting that the differences were not caused by background methylation. Considering that the left and right colons manifested significant differences in tissue origin, cancer pathogenesis, and clinical prognosis [48], it is easy to interpret that SDC2 was differentially methylated among the different locations. Owing to the distinctiveness that most of the CRC cases were left‐sided in China [49], there would be a serious impact on the overall detection rate of CRC if the screening for SDC2 methylation was inadequate. However, one limitation of this study was that the different methylation levels of tumor locations and their impact on CRC detection require more thorough experimental evidence to be validated, such as the performance of stool samples derived from CRC patients, which has already been initiated in our laboratory.

In this study, we verified the feasibility of using two methylated fragments in the SDC2 CpG islands as detection targets. The results indicated that a panel of multiple targets could improve the detection sensitivity of CRC, which might potentially improve the detection of left‐sided colon cancers. Further efforts to evaluate other biomarkers with better complementary effects are imperative, considering the difference in methylation levels between right‐sided and left‐sided tumors.

## Conflicts of interest

The authors declare no conflict of interest.

## Author contributions

Conceptualization, RL, BQ, JL, and FZ; methodology, RL; software, BQ; validation, RL, KW, and CL; formal analysis, RL; investigation, TL; resources, RL; data curation, RL, KW; writing—original draft preparation, RL; writing—review and editing, FZ; visualization, JL; supervision, FZ; project administration, FZ; funding acquisition, FZ. All authors have read and agreed to the published version of the manuscript.

## Supporting information

Table S1. Clinical characters of TCGA CRC samples.
Table S2. The CpG island information of SDC2.
Table S3. Probes information within the CpG island.
Table S4. Sensitivity and specificity of 5 DMRs estimated by TCGA CRC data.
Table S5. Sensitivity and specificity of MSPR1 and MSPR2 estimated by 252 tissue samples.

## Data Availability

The TCGA CRC 450k data were public access available online (https://portal.gdc.cancer.gov/). The MSP data used and analyzed in this study are available from the corresponding author on reasonable request.

## References

[1] Sung H , Ferlay J , Siegel RL , Laversanne M , Soerjomataram I , Jemal A and Bray F (2021) Global cancer statistics 2020: GLOBOCAN estimates of incidence and mortality worldwide for 36 cancers in 185 countries. CA Cancer J Clin 71, 209–249. 33538338 10.3322/caac.21660

[2] Chen W , Zheng R , Baade PD , Zhang S , Zeng H , Bray F , Jemal A , Yu XQ and He J . (2016) Cancer statistics in China, 2015. CA Cancer J Clin 66, 115–132. 26808342 10.3322/caac.21338

[3] Marković BB (2015) Prevention and early detection of colorectal cancer. Acta Med Croatica 69, 365–371. 29083907

[4] Chen HD , Li N , Ren JS , Shi JF , Zhang YM , Zou SM , Zheng ZX , Zhang K and Dai M (2018) Compliance rate of screening colonoscopy and its associated factors among high‐risk populations of colorectal cancer in urban China. Zhonghua Yu Fang Yi Xue Za Zhi 52, 231–237. 29973000 10.3760/cma.j.issn.0253-9624.2018.03.004

[5] Lee JK , Liles EG , Bent S , Levin TR and Corley DA (2014) Accuracy of fecal immunochemical tests for colorectal cancer: systematic review and meta‐analysis. Ann Intern Med 160, 171. 24658694 10.7326/M13-1484 PMC4189821

[6] Grady WM and Pritchard CC (2014) Molecular alterations and biomarkers in colorectal cancer. Toxicol Pathol 42, 124–139. 24178577 10.1177/0192623313505155 PMC3947043

[7] Reik W , Dean W and Walter J (2001) Epigenetic reprogramming in mammalian development. Science 293, 1089–1093. 11498579 10.1126/science.1063443

[8] Meng H , Cao Y , Qin J , Song X , Zhang Q , Shi Y and Cao L (2015) DNA methylation, its mediators and genome integrity. Int J Biol Sci 11, 604–617. 25892967 10.7150/ijbs.11218 PMC4400391

[9] Morgan AE , Davies TJ and Mc AM (2018) The role of DNA methylation in ageing and cancer. Proc Nutr Soc 77, 412–422. 29708096 10.1017/S0029665118000150

[10] Wu SC and Zhang Y (2010) Active DNA demethylation: many roads lead to Rome. Nat Rev Mol Cell Biol 11, 607–620. 20683471 10.1038/nrm2950 PMC3711520

[11] Kulis M and Esteller M (2010) DNA methylation and cancer. Adv Genet 70, 27–56. 20920744 10.1016/B978-0-12-380866-0.60002-2

[12] Johnson DA , Barclay RL , Mergener K , Weiss G , König T , Beck J and Potter NT (2014) Plasma Septin9 versus fecal immunochemical testing for colorectal cancer screening: a prospective multicenter study. PLoS ONE 9, e98238. 24901436 10.1371/journal.pone.0098238 PMC4046970

[13] Imperiale TF , Ransohoff DF , Itzkowitz SH , Levin TR , Lavin P , Lidgard GP , Ahlquist DA and Berger BM (2014) Multitarget stool DNA testing for colorectal‐cancer screening. N Engl J Med 370, 1287–1297. 24645800 10.1056/NEJMoa1311194

[14] Mojtabanezhad Shariatpanahi A , Yassi M , Nouraie M , Sahebkar A , Varshoee Tabrizi F and Kerachian MA (2018) The importance of stool DNA methylation in colorectal cancer diagnosis: A meta‐analysis. PLoS ONE 13, e200735. 10.1371/journal.pone.0200735 PMC6053185 30024936

[15] Patai ÁV , Valcz G , Hollósi P , Kalmár A , Péterfia B , Patai Á , Wichmann B , Spisák S , Barták BK , Leiszter K *et al*. (2015) Comprehensive DNA methylation analysis reveals a common ten‐gene methylation signature in colorectal adenomas and carcinomas. PLoS ONE 10, e133836. 10.1371/journal.pone.0133836 PMC4546193 26291085

[16] Mitchell SM , Ross JP , Drew HR , Ho T , Brown GS , Saunders NFW , Duesing KR , Buckley MJ , Dunne R , Beetson I *et al*. (2014) A panel of genes methylated with high frequency in colorectal cancer. BMC Cancer 14, 54. 24485021 10.1186/1471-2407-14-54 PMC3924905

[17] Oh T , Kim N , Moon Y , Kim MS , Hoehn BD , Park CH , Kim TS , Kim NK , Chung HC and An S (2013) Genome‐wide identification and validation of a novel methylation biomarker, SDC2, for blood‐based detection of colorectal cancer. J Mol Diagn 15, 498–507. 23747112 10.1016/j.jmoldx.2013.03.004

[18] Park H , Kim Y , Lim Y , Han I and Oh ES (2002) Syndecan‐2 mediates adhesion and proliferation of colon carcinoma cells. J Biol Chem 277, 29730–29736. 12055189 10.1074/jbc.M202435200

[19] Han YD , Oh TJ , Chung T , Jang HW , Kim YN , An S and Kim NK (2019) Early detection of colorectal cancer based on presence of methylated syndecan‐2 (SDC2) in stool DNA. Clin Epigenetics 11, 51. 30876480 10.1186/s13148-019-0642-0 PMC6419806

[20] Niu F , Wen J , Fu X , Li C , Zhao R , Wu S , Yu H , Liu X , Zhao X , Liu S *et al*. (2017) Stool DNA test of methylated syndecan‐2 for the early detection of colorectal neoplasia. Cancer Epidemiol Biomarkers Prev 26, 1411. 28619831 10.1158/1055-9965.EPI-17-0153

[21] Koch A , Joosten SC , Feng Z , de Ruijter TC , Draht MX , Melotte V , Smits KM , Veeck J , Herman JG , Van Neste L *et al*. (2018) Analysis of DNA methylation in cancer: location revisited. Nat Rev Clin Oncol 15, 459–466. 29666440 10.1038/s41571-018-0004-4

[22] Homma N , Tamura G , Honda T , Matsumoto Y , Nishizuka S , Kawata S and Motoyama T (2006) Spreading of methylation within RUNX3 CpG island in gastric cancer. Cancer Sci 97, 51–56. 16367921 10.1111/j.1349-7006.2005.00133.x PMC11159484

[23] Deng G , Chen A , Hong J , Chae HS and Kim YS (1999) Methylation of CpG in a small region of the hMLH1 promoter invariably correlates with the absence of gene expression. Cancer Res 59, 2029. 10232580

[24] Hodges E , Smith AD , Kendall J , Xuan Z , Ravi K , Rooks M , Zhang MQ , Ye K , Bhattacharjee A , Brizuela L *et al*. (2009) High definition profiling of mammalian DNA methylation by array capture and single molecule bisulfite sequencing. Genome Res 19, 1593–1605. 19581485 10.1101/gr.095190.109 PMC2752124

[25] Martorell‐Marugán J , González‐Rumayor V and Carmona‐Sáez P (2019) mCSEA: detecting subtle differentially methylated regions. Bioinformatics 35, 3257–3262. 30753302 10.1093/bioinformatics/btz096

[26] Kolde R , Märtens K , Lokk K , Laur S and Vilo J (2016) seqlm: an MDL based method for identifying differentially methylated regions in high density methylation array data. Bioinformatics 32, 2604–2610. 27187204 10.1093/bioinformatics/btw304 PMC5013909

[27] Peters TJ , Buckley MJ , Statham AL , Pidsley R , Samaras K , V Lord R , Clark SJ and Molloy PL (2015) De novo identification of differentially methylated regions in the human genome. Epigenetics Chromatin 8, 6. 25972926 10.1186/1756-8935-8-6 PMC4429355

[28] Jaffe AE , Murakami P , Lee H , Leek JT , Fallin MD , Feinberg AP and Irizarry RA (2012) Bump hunting to identify differentially methylated regions in epigenetic epidemiology studies. Int J Epidemiol 41, 200–209. 22422453 10.1093/ije/dyr238 PMC3304533

[29] Lent S , Xu H , Wang L , Wang Z , Sarnowski C , Hivert MF and Dupuis J (2018) Comparison of novel and existing methods for detecting differentially methylated regions. BMC Genet 19, 84. 30255775 10.1186/s12863-018-0637-4 PMC6156895

[30] Koch A , De Meyer T , Jeschke J and Van Criekinge W (2015) MEXPRESS: visualizing expression, DNA methylation and clinical TCGA data. BMC Genom 16, 636. 10.1186/s12864-015-1847-z PMC4549898 26306699

[31] Wang Z , Li X , Jiang Y , Shao Q , Liu Q , Chen B and Huang D (2015) swDMR: a sliding window approach to identify differentially methylated regions based on whole genome bisulfite sequencing. PLoS ONE 10, e132866. 10.1371/journal.pone.0132866 PMC4503785 26176536

[32] Oh TJ , Oh HI , Seo YY , Jeong D , Kim C , Kang HW , Han YD , Chung HC , Kim NK and An S (2017) Feasibility of quantifying SDC2 methylation in stool DNA for early detection of colorectal cancer. Clin Epigenetics 9, 126. 29225717 10.1186/s13148-017-0426-3 PMC5715626

[33] Ooki A , Maleki Z , Tsay JJ , Goparaju C , Brait M , Turaga N , Nam H , Rom WN , Pass HI , Sidransky D *et al*. (2017) A panel of novel detection and prognostic methylated DNA markers in primary non‐small cell lung cancer and serum DNA. Clin Cancer Res 23, 7141. 28855354 10.1158/1078-0432.CCR-17-1222

[34] Nawaz I , Qiu X , Wu H , Li Y , Fan Y , Hu L , Zhou Q and Ernberg I (2014) Development of a multiplex methylation specific PCR suitable for (early) detection of non‐small cell lung cancer. Epigenetics 9, 1138–1148. 24937636 10.4161/epi.29499 PMC4164499

[35] Wu D , Zhou G , Jin P , Zhu J , Li S , Wu Q , Wang G , Sheng J , Wang J , Song L *et al*. (2016) Detection of colorectal cancer using a simplified SEPT9 gene methylation assay is a reliable method for opportunistic screening. J Mol Diagn 18, 535–545. 27133379 10.1016/j.jmoldx.2016.02.005

[36] R Core Team (2018) R: A language and environment for statistical computing. R Foundation for Statistical Computing, Vienna, Austria. https://www.R‐project.org/

[37] Robin X , Turck N , Hainard A , Tiberti N , Lisacek F , Sanchez J and Müller M (2011) pROC: an open‐source package for R and S+ to analyze and compare ROC curves. BMC Bioinformatics 12, 77. 21414208 10.1186/1471-2105-12-77 PMC3068975

[38] Friedman JH , Hastie T and Tibshirani R (2010) Regularization paths for generalized linear models via coordinate descent. J Stat Softw 1, 2010. PMC2929880 20808728

[39] Bibikova M , Barnes B , Tsan C , Ho V , Klotzle B , Le JM , Delano D , Zhang L , Schroth GP , Gunderson KL *et al*. (2011) High density DNA methylation array with single CpG site resolution. Genomics 98, 288–295. 21839163 10.1016/j.ygeno.2011.07.007

[40] Nazemalhosseini ME , Kuppen PJ , Aghdaei HA and Zali MR (2013) The CpG island methylator phenotype (CIMP) in colorectal cancer. Gastroenterol Hepatol Bed Bench 6, 120–128. 24834258 PMC4017514

[41] Missiaglia E , Jacobs B , D'Ario G , Di Narzo AF , Soneson C , Budinska E , Popovici V , Vecchione L , Gerster S , Yan P *et al*. (2014) Distal and proximal colon cancers differ in terms of molecular, pathological, and clinical features. Ann Oncol 25, 1995–2001. 25057166 10.1093/annonc/mdu275

[42] Bauer KM , Hummon AB and Buechler S (2012) Right‐side and left‐side colon cancer follow different pathways to relapse. Mol Carcinog 51, 411–421. 21656576 10.1002/mc.20804 PMC4104680

[43] Galamb O , Kalmár A , Barták BK , Patai ÁV , Leiszter K , Péterfia B , Wichmann B , Valcz G , Veres G , Tulassay Z *et al*. (2016) Aging related methylation influences the gene expression of key control genes in colorectal cancer and adenoma. World J Gastroenterol 22, 10325–10340. 28058013 10.3748/wjg.v22.i47.10325 PMC5175245

[44] Johnson AA , Akman K , Calimport SR , Wuttke D , Stolzing A and de Magalhães JP (2012) The role of DNA methylation in aging, rejuvenation, and age‐related disease. Rejuvenation Res 15, 483–494. 23098078 10.1089/rej.2012.1324 PMC3482848

[45] Kwon MJ , Kim Y , Choi Y , Kim SH , Park S , Han I , Kang DH and Oh ES (2013) The extracellular domain of syndecan‐2 regulates the interaction of HCT116 human colon carcinoma cells with fibronectin. Biochem Biophys Res Commun 431, 415–420. 23333331 10.1016/j.bbrc.2012.12.155

[46] Miranda TB and Jones PA (2007) DNA methylation: the nuts and bolts of repression. J Cell Physiol 213, 384–390. 17708532 10.1002/jcp.21224

[47] Dedeurwaerder S , Defrance M , Calonne E , Denis H , Sotiriou C and Fuks F (2011) Evaluation of the infinium methylation 450K technology. Epigenomics 3, 771–784. 22126295 10.2217/epi.11.105

[48] Meza R , Jeon J , Renehan AG and Luebeck EG (2010) Colorectal cancer incidence trends in the United States and United kingdom: evidence of right‐ to left‐sided biological gradients with implications for screening. Cancer Res 70, 5419–5429. 20530677 10.1158/0008-5472.CAN-09-4417 PMC2914859

[49] Li M and Gu J (2005) Changing patterns of colorectal cancer in China over a period of 20 years. World J Gastroenterol 11, 4685–4688. 16094710 10.3748/wjg.v11.i30.4685 PMC4615411

